# Magnetic resonance-guided focused ultrasound thalamotomy for essential tremor patients with low skull density ratio: a case-matched analysis

**DOI:** 10.3389/fneur.2024.1370574

**Published:** 2024-04-22

**Authors:** Patrick R. Ng, Sarah E. Blitz, Melissa M. J. Chua, G. Rees Cosgrove

**Affiliations:** ^1^Department of Neurological Surgery, University of Southern California, Keck School of Medicine, Los Angeles, CA, United States; ^2^Harvard Medical School, Boston, CA, United States; ^3^Department of Neurosurgery, Brigham and Women’s Hospital, Boston, MA, United States

**Keywords:** focused ultrasound, thalamotomy, essential tremor, skull density ratio, case match

## Abstract

**Introduction:**

Skull density ratio (SDR) is the ratio between the mean Hounsfield units of marrow and cortical bone, impacting energy transmission through the skull. Low SDR has been used as an exclusion criterion in major trials of magnetic resonance-guided focused ultrasound (MRgFUS) thalamotomy for medication-refractory essential tremor (ET). However, some studies have suggested that patients with low SDR can safely undergo MRgFUS with favorable outcomes. In this case-matched study, we aim to compare the characteristics, sonication parameters, lesion sizes, and clinical outcomes of patients with low SDR vs. patients with high SDR who underwent unilateral MRgFUS thalamotomy for medication-refractory ET.

**Methods:**

Between March 2016 and April 2023, all patients (*n* = 270) who underwent unilateral MRgFUS thalamotomy for medication-refractory ET at a single institution were classified as low SDR (<0.40) and high SDR (≥0.40). All clinical and radiological data was prospectively collected and retrospectively analyzed using non-case-matched and 1:1 case-matched methodology.

**Results:**

Thirty-one patients had low SDR, and 239 patients had high SDR. Fifty-six patients (28 in each cohort) were included in 1:1 case-matched analysis. There were no significant differences in baseline characteristics between the two groups in both non-case-matched and 1:1 case-matched analyses. In both analyses, compared to patients with high SDR, patients with low SDR required a significantly higher maximum sonication power, energy, and duration, and reached a lower maximum temperature with smaller lesion volumes. In the non-case-matched and case-matched analyses, low SDR patients did not have significantly less tremor control at any postoperative timepoints. However, there was a higher chance of procedure failure in the low SDR group with three patients not obtaining an appropriately sized lesion. In both analyses, imbalance was observed more often in high SDR patients on postoperative day 1 and month 3.

**Discussion:**

ET patients with SDR <0.40 can be safely and effectively treated with MRgFUS, though there may be higher rates of treatment failure and intraoperative discomfort.

## 1 Introduction

Magnetic resonance-guided focused ultrasound (MRgFUS) thalamotomy is an FDA-approved, minimally invasive therapy for the treatment of medication refractory Essential Tremor (ET) (1, 2). With MRgFUS thalamotomy, high-intensity ultrasound beams are focused on the ventralis intermedius nucleus (VIM), creating a thermal lesion that has been shown to reduce pathological tremors (3). In the pivotal, randomized, sham-controlled trial that led to FDA-approval of MRgFUS thalamotomy for ET, hand tremor was improved by 47% at 3 months, and clinical benefits were sustained at 5 years with no progressive or delayed complications (4–6). Patient outcomes with MRgFUS have continued to improve in more recent trials (7).

Among several preoperative criteria used to select patients for MRgFUS, skull density ratio (SDR) has been one of the most widely applied and debated (8). SDR is defined as the ratio between the mean values (in Hounsfield units) of marrow and cortical bone as measured by preoperative computed tomography (CT) scans (9). Lower SDR has been postulated to interfere with transcranial energy transmission via greater attenuation and reflection of ultrasonic energy at the marrow/cortical bone interface (9). Indeed, clinical studies have shown that SDR affects energy delivery and efficiency (9, 10). Based in part on the inclusion criteria of the pivotal trial (4), the FDA has established an SDR of 0.45 (±0.05) or less as a contraindication for MRgFUS (1). While SDR is an important factor in determining technical feasibility of MRgFUS, some studies have demonstrated no significant associations between SDR and clinical outcomes at one year follow-up (8, 11–13). Additionally, while SDR may impact the ability to reach high maximum temperatures, multiple lower-temperature sonications have been demonstrated to reach a high enough accumulated thermal dose to create an appropriate lesion (14). Furthermore, patients with SDR <0.45 may represent 30–40% of ET patients who could potentially benefit from MRgFUS, especially East Asian patients who tend to have lower SDRs (15–17). The FDA’s SDR cutoff of 0.45 (±0.05) may therefore exclude a significant proportion of patients who could benefit from an effective therapy with a growing number of indications (18).

A recent report by Vetkas et al. (19) analyzed differences in MRgFUS in patients with low SDR and high SDR, but very few patients had follow up of tremor scores and adverse events. In our large patient population, we aimed to better characterize these differences and complete a case-matched cohort analysis to more directly understand the effect of SDR on tremor outcomes. We report a study comparing the characteristics, sonication parameters, lesion sizes, and clinical outcomes of patients with low SDR vs. patients with high SDR and present one illustrative case.

## 2 Methods

This case-matched cohort study was designed to compare the clinical characteristics, sonication parameters, lesion size, and tremor outcomes of patients with low SDR vs. patients with high SDR. This study was conducted at a single center (Brigham and Women’s Hospital, Boston, MA, United States) with local institutional review board approval.

### 2.1 Patient selection

Between March 2016 and April 2023, all patients (*n* = 270) who underwent unilateral MRgFUS thalamotomy for medication-refractory ET had their clinical and radiological data prospectively collected. All patients with an SDR < 0.40 were assigned to the low-SDR cohort (*n* = 31). For non-case-matched analyses, all remaining patients were assigned to the high-SDR cohort (*n* = 239). Patients who did not achieve a goal lesion of at least 4 mm (*n* = 3) were excluded from analyses of sonication parameters, tremor control, side effects, and lesion volume. For a supplemental analysis, patients were also grouped into low (< 0.40), medium (≥ 0.40, < 0.60), and high (≥ 0.60) SDR groups, and tremor control and sonication parameters between these three groups were compared. For case-matched analyses, we conducted a 1:1 matching between low-SDR and high-SDR cohorts with the following variables: age (within 2 years), sex, and date of procedure (within 7 months) (*n* = 28 in each cohort).

### 2.2 Prospective database

The following variables were prospectively collected for every patient: demographics (age, sex, handedness), disease characteristics (family history, tremor duration, baseline tremor scores), SDR, treatment laterality, presence of intraoperative side effects, sonication parameters (sonication number, maximum power, maximum energy, number of sonications with energy >5000 J, maximum sonication duration, maximum temperature), follow-up tremor scores, follow-up percent improvement in tremor scores relative to baseline scores, and adverse events (fatigue, weakness, dysarthria, dysgeusia, sensory changes including numbness and/or paresthesia, and imbalance).

Tremor scores were measured using the Fahn-Tolosa-Marin (FTM) scale (20). We report total FTM score, which is a composite score of the following categories with 0 to 4 points (no tremor – 0; slight tremor – 1; moderate tremor – 2; marked tremor – 3; severe tremor – 4) assigned to each category, yielding a maximum total score of 20: vocal tremor, head tremor, resting tremor of the affected limb, intention tremor of the affected limb, and postural tremor of the affected limb. We also report intention + posture FTM score, which combines 0-to-4-point scores for intention and postural tremors of the affected limb, yielding a maximum total score of 8. Tremor scores were recorded at baseline and postoperatively on day 1, 3 months, 1 year, and each annual follow-up thereafter. Adverse events were also documented at these follow-up timepoints. Not all patients had follow-up data at all timepoints. Only available data was included in analyses at each timepoint.

### 2.3 Procedure

The procedural workflow at our institution has been previously reported (21). In brief, all patients underwent preoperative CT scans to measure SDR. On the day of treatment, the patient’s head was shaved, and a modified Cosman-Roberts Wells frame (Radionics, Inc.) was secured low enough on the patient’s head to accommodate the silicone membrane associated with the ExAblate system. The patient was positioned on a 3T magnetic resonance imaging (MRI) table (GE Medical Systems) and connected to the ExAblate 4000 MRgFUS hemispheric transducer operating at 650 Hz (InSightec, Inc.). The space between the patient’s head and the transducer was then filled with cooled, degassed water. Baseline MRI sequences were obtained to assist with indirect targeting via standardized stereotactic coordinates and anatomical landmarks. Initial target coordinates for the VIM were set at 25% of the anterior commissure-posterior commissure (AC-PC) distance anterior to the PC, 1.5–2 mm superior to the AC-PC plane, and 14 mm lateral to the midline or 11 mm lateral to the wall of the third ventricle. Low-energy test sonications were delivered under MR thermometry guidance to confirm appropriate alignment. With confirmed targeting, high-energy sonications were delivered sequentially to a maximum temperature goal of 55–60°C. Clinical exams were conducted after each treatment to monitor for side effects and tremor improvement.

### 2.4 Lesion analysis

Thin-cut (2 mm) axial and coronal T2-weighted MRI slices were obtained within 24 h postoperatively. Lesions were manually segmented in 3D slicer.^1^ A lesion was defined as combined Wintermark zones 1 and 2, which represent coagulative necrosis and cytotoxic edema, respectively (22). Segmented lesion volume data was analyzed in MATLAB 2022a (The MathWorks, Inc., Natick, Massachusetts, United States). Two patients did not have a T2-weighted MRI at 24 h and were therefore excluded from volumetric analyses.

### 2.5 Statistical analysis

Statistical analyses were conducted within Microsoft Excel (Microsoft Corporation, Redmond, WA), and Python version 3 (Python Software Foundation, Fredericksburg, VA). Continuous variables were reported as mean (± SD) or median (range) and were analyzed with independent t-tests, while categorical variables were analyzed with Chi-squared tests. Statistical significance was set at *p* < 0.05.

## 3 Results

### 3.1 Non-case-matched analysis

Overall, thirty-one patients had low SDR (i.e., SDR < 0.40), and two hundred and thirty-nine patients had high SDR. Median (range) SDRs in the low-SDR and high-SDR groups were 0.36 (0.32–0.39) and 0.49 (0.40–0.76), respectively. There were no significant differences between the two groups in the following characteristics: sex, handedness, duration of tremor, total preoperative FTM score, intention + posture preoperative FTM score, age at treatment, treatment laterality, and rate of intraoperative side effects (Table 1).

**Table 1:** Characteristics of low SDR vs. high SDR patients in the non-case-matched analysis.

Variable	Low SDR	High SDR	*p* value
Number of patients	31	239	
SDR (mean ± SD)	0.36 ± 0.02	0.51 ± 0.08	
Sex (females) (%)	41.9	31.8	1.0
Dominant hand (right) (%)	93.6	81.6	1.0
Duration of tremor (years) (mean ± SD)	23.8 ± 18.1	28.0 ± 18.5	0.292
Preop FTM score (total) (mean ± SD)	7.1 ± 2.0	7.0 ± 2.3	0.773
Preop FTM score (intention + posture) (mean ± SD)	5.9 ± 1.2	5.8 ± 1.2	0.680
Age at treatment (mean ± SD)	76.0 ± 8.1	74.7 ± 7.0	0.361
Treatment laterality (left) (%)	83.9	78.7	1.0
Intraoperative side effects (%)	6.5	9.2	1.0
3 month postop FTM score (intention + posture) (mean ± SD)	0.6 ± 1.1 (*n* = 19)	0.4 ± 1.0 (*n* = 176)	0.30
1 year postop FTM score (intention + posture) (mean ± SD)	0.9 ± 2.0 (*n* = 15)	0.8 ± 1.5 (*n* = 156)	0.73

Continuous variables were analyzed with independent *t*-tests, while categorical variables were analyzed with Chi-squared tests. Significance was set at *p* < 0.05.

**Figure 1:**
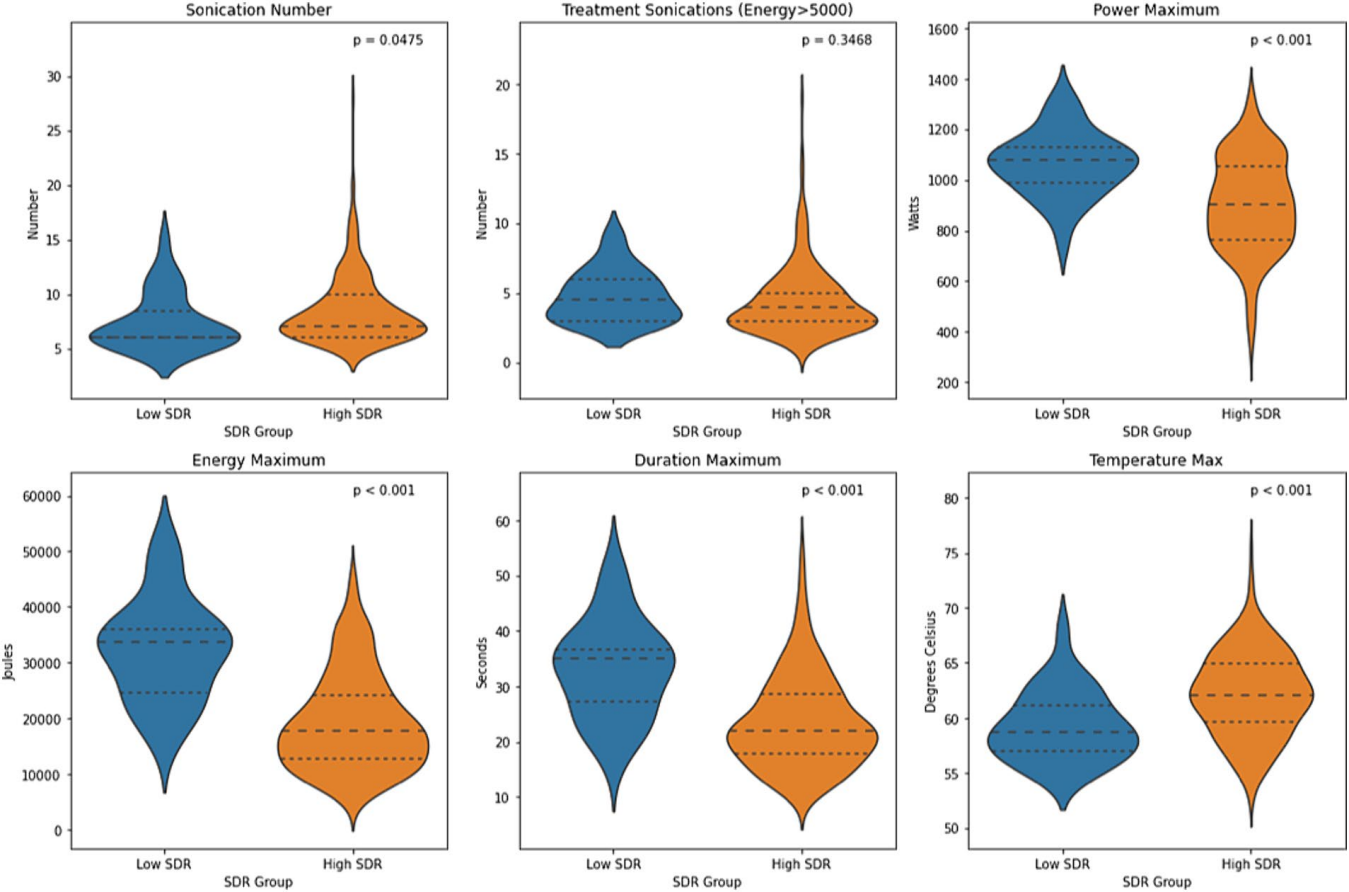
Violin plots of sonication number, treatment sonications with energy >5000 J, mean maximum power, mean maximum energy, mean maximum sonication duration, and mean maximum temperature between low-SDR (*n* = 28) and high-SDR (*n* = 239) cohorts. Continuous variables were analyzed with independent *t*-tests. Significance set at *p* < 0.05. SDR: skull density ratio.

Except for number of treatment sonications with energy >5000 J, all other sonication parameters, including sonication number, maximum power, maximum energy, maximum duration, and maximum temperature, differed significantly between low-SDR and high-SDR cohorts (Figure 1). Patients with low SDR required a lower sonication number (*p* < 0.05) and a higher maximum power (*p* < 0.001), energy (*p* < 0.001), and duration (*p* < 0.001), and reached a lower maximum temperature (*p* < 0.001). There was a significant difference in the mean lesion volumes (± SD) with low-SDR patients demonstrating a smaller lesion volume (302.5 ± 150.4 mm^3^, *n* = 28) than high-SDR patients (435.8 ± 185.9 mm^3^, *n* = 237) (*p* = 0.0003).

**Figure 2:**
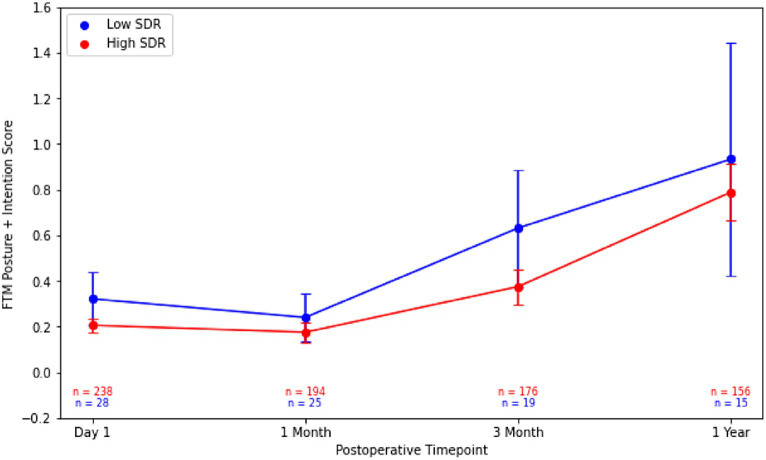
Post-operative Fahn-Tolosa-Marin (FTM) intention + posture scores over time after MRgFUS for essential tremor comparing all patients with low SDR (*n* = 28) to all patients with high SDR (*n* = 239). Continuous variables were analyzed with independent *t*-tests. Significance set at *p* < 0.05. SDR: skull density ratio.

**Figure 3:**
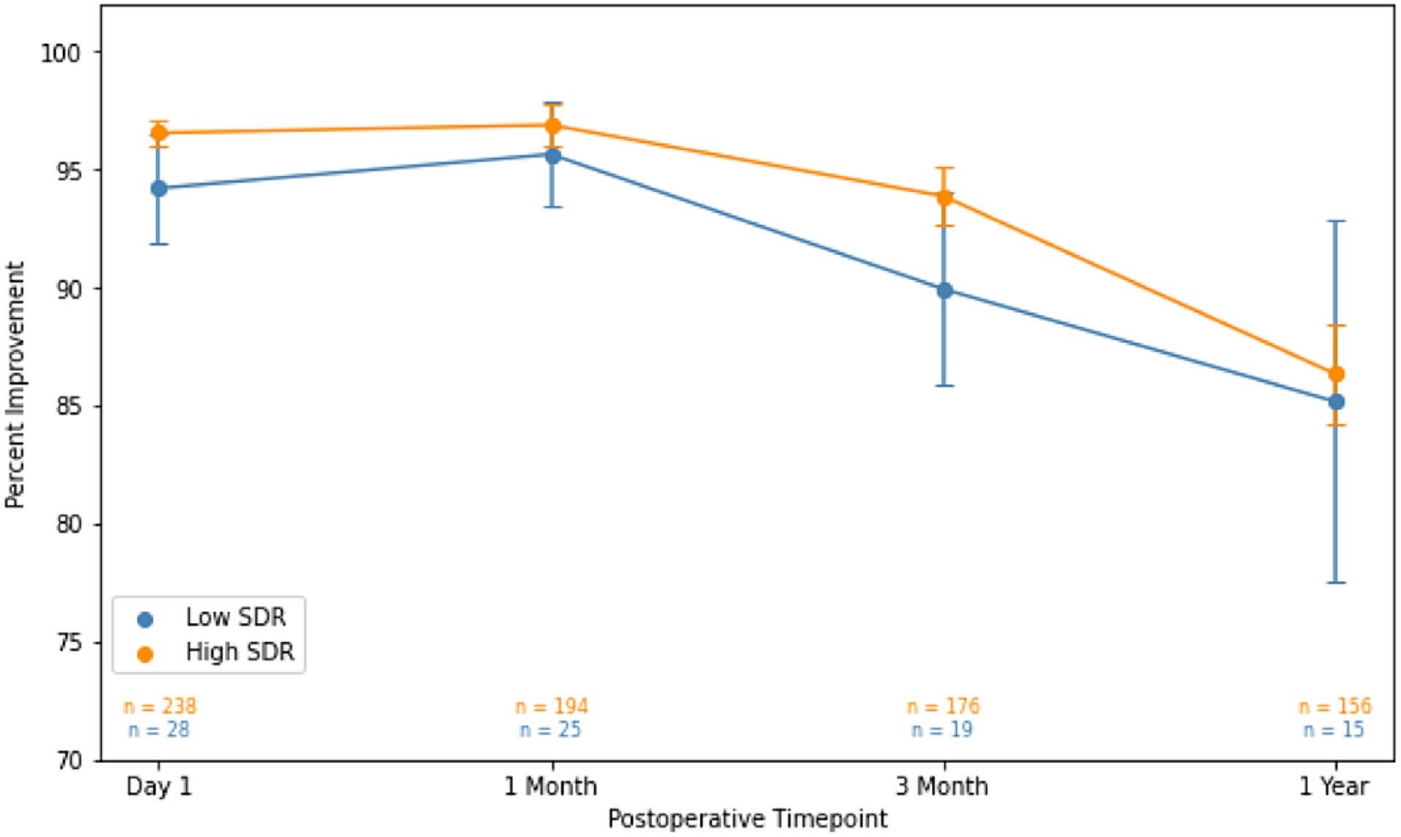
Percent improvement in essential tremor (intention + posture FTM scores) for patients at various timepoints after MRgFUS for essential tremor comparing all patients with low SDR (*n* = 28) to all patients with high SDR (*n* = 239).Populations compared using independent *t*-tests. Significance set at *p* < 0.05. SDR: skull density ratio.

Although patients with low SDR tended to have lower tremor control at all timepoints, there were no significant differences in the absolute intention + posture FTM scores and % improvement in intention + posture FTM scores relative to baseline between low-SDR and high-SDR patients (Figures 2, 3). Regarding adverse events, there were significant differences between low-SDR and high-SDR cohorts in postoperative day 1 imbalance (low-SDR: 32.1%; high-SDR: 66.0%; *p* = 0.002) and postoperative month 3 imbalance (low-SDR: 5.6%; high-SDR: 31.8%; *p* = 0.04) (Table 2).

**Table 2:** Side effects over time after MRgFUS for essential tremor comparing all patients with low SDR (*n* = 28) to patients with high SDR (*n* = 239).

	%	Overall	Weakness	Sensory	Dysarthria	Imbalance	Dysmetria/Discoordination	Dysgeusia
Day 1	Low SDR (*n* = 28)	57.1	3.6	25.0	7.1	32.1	3.6	3.6
	High SDR (*n* = 238)	76.9	11.3	26.1	16.8	66.0	15.1	2.1
	*p*-value	0.030	0.244	0.700	0.180	0.002	0.104	1.000
1 Month	Low SDR (*n* = 25)	88.0	4.0	32.0	8.0	64.0	12.0	24.0
	High SDR (*n* = 194)	86.1	17.0	32.5	15.5	57.2	31.4	13.4
	*p*-value	1.00	0.146	1.000	0.448	0.836	0.062	0.309
3 Months	Low SDR (*n* = 18)	38.9	0	22.2	0	5.6	5.6	11.1
	High SDR (*n* = 176)	60.2	6.2	27.8	4.5	31.8	14.8	10.8
	*p*-value	0.134	0.577	0.817	0.763	0.040	0.472	1.000
1 Year	Low SDR (*n* = 15)	20.0	0	6.7	0	6.7	13.3	0
	High SDR (*n* = 156)	42.3	3.8	15.4	4.5	18.6	10.3	7.1
	*p*-value	0.160	0.969	0.596	0.876	0.421	1.000	0.608

Categorical variables were analyzed with Chi-squared tests. Significance was set at *p* < 0.05.

An analysis comparing patients with low (< 0.40), medium (≥ 0.40, < 0.60), and high (≥ 0.60) SDR showed that all of the differences in sonication parameters are graded (Supplementary Figure S1). When looking at tremor control, patients with medium and high SDR had extremely similar outcomes (Supplementary Figure S2). These groups both tended to have better percent improvement than those with low SDR, but there were no statistically significant differences.

### 3.2 Case-matched analysis

The low-SDR and high-SDR case-matched cohorts included twenty-eight patients each. Median (range) SDR in the low-SDR and high-SDR groups were 0.36 (0.32–0.39) and 0.48 (0.41–0.67), respectively. There were no significant differences between the two groups in the following characteristics: sex, handedness, duration of tremor, total preoperative FTM score, intention + posture preoperative FTM score, age at treatment, treatment laterality, and rate of intraoperative side effects (Table 3).

**Table 3:** Characteristics of low SDR vs. high SDR patients in the case-matched analysis.

Variable	Low SDR	High SDR	*p* value
Number of patients	28	28	
SDR (mean ± SD)	0.36 ± 0.02	0.50 ± 0.06	
Sex (females) (%)	42.9	42.9	1.0
Dominant hand (right) (%)	89.3	82.1	1.0
Duration of tremor (years) (mean ± SD)	21.3 ± 16.7	27.9 ± 19.5	0.29
Preop FTM score (total) (mean ± SD)	6.89 ± 1.57	7.00 ± 2.52	0.85
Preop FTM score (intention + posture) (mean ± SD)	6.00 ± 1.28	5.82 ± 1.28	0.60
Age at treatment (mean ± SD)	74.4 ± 6.9	74.2 ± 6.6	0.92
Treatment laterality (left) (%)	78.6	75.0	1.0
Intraoperative side effects (%)	0.0	7.1	1.0
3 month postop FTM score (intention + posture) (mean ± SD)	0.4 ± 0.8 (*n* = 15)	0.8 ± 1.7 (*n* = 15)	0.42
1 year postop FTM score (intention + posture) (mean ± SD)	1.3 ± 2.4 (*n* = 10)	0.5 ± 1.1 (*n* = 10)	0.34

Continuous variables were analyzed with independent *t*-tests, while categorical variables were analyzed with Chi-squared tests. Significance was set at *p* < 0.05.

**Figure 4:**
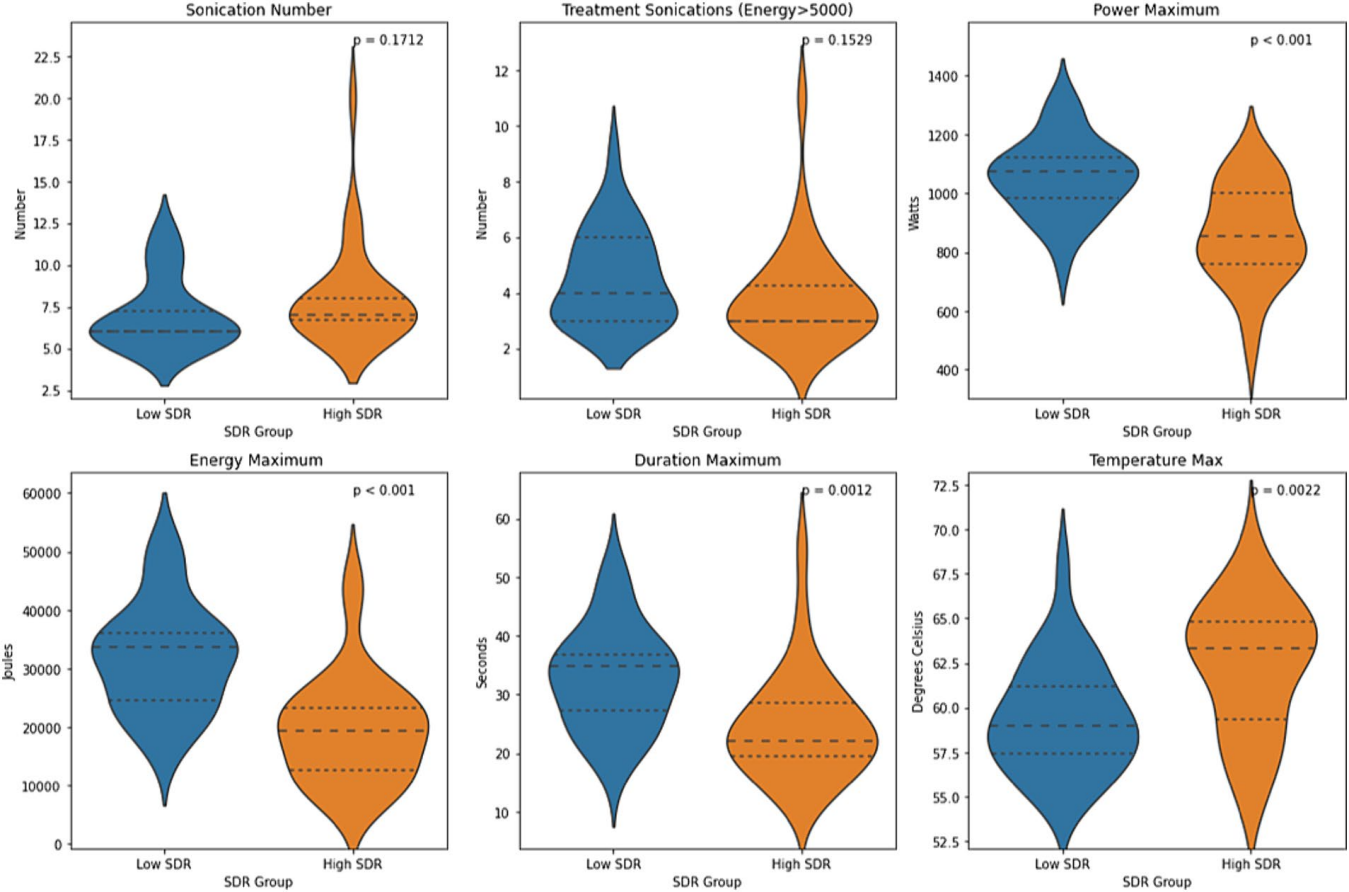
Violin plots of sonication number, treatment sonications with energy >5000 J, mean maximum power, mean maximum energy, mean maximum sonication duration, and mean maximum temperature between case-matched low-SDR (*n* = 28) and high-SDR (*n* = 28) cohorts. Continuous variables were analyzed with independent *t*-tests. Significance set at *p* < 0.05. SDR: skull density ratio.

Except for sonication number and number of treatment sonications with energy >5000 J, all other sonication parameters, including maximum power, maximum energy, maximum duration, and maximum temperature, differed significantly between low-SDR and high-SDR cohorts (Figure 4). Patients with low SDR required a higher maximum power (*p* < 0.001), energy (*p* < 0.001), and duration (*p* = 0.001), and reached a lower maximum temperature (*p* = 0.002). There was a significant difference in the mean lesion volumes (± SD) between low-SDR patients (293.7 ± 153.7 mm^3^, *n* = 28) and high-SDR patients (433.7 ± 265.8 mm^3^, *n* = 28) (*p* = 0.02).

**Figure 5:**
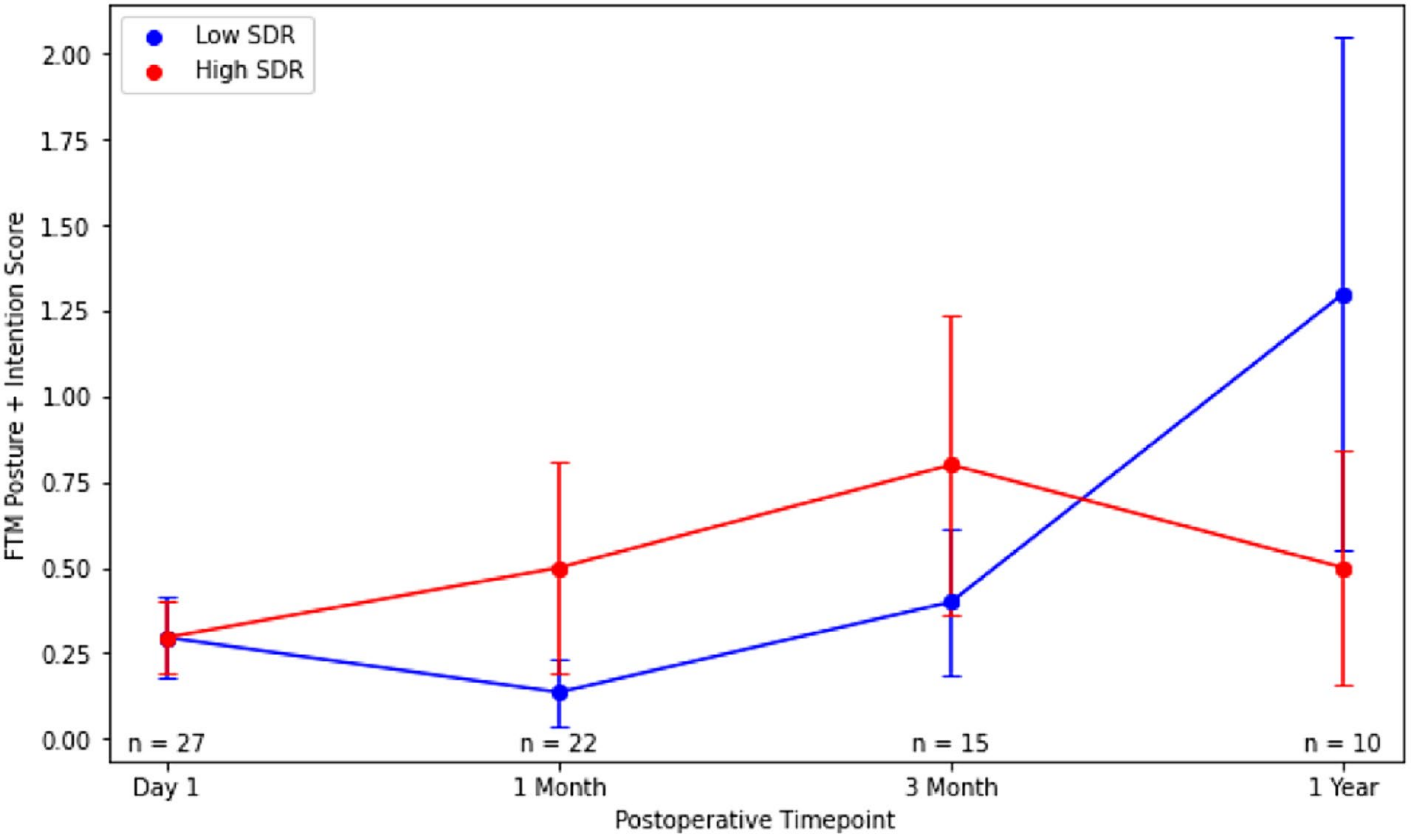
Post-operative Fahn-Tolosa-Marin (FTM) intention + posture scores over time after MRgFUS for essential tremor comparing case-matched cohorts with low SDR (*n* = 28) and high SDR (*n* = 28). Continuous variables were analyzed with independent *t*-tests. Significance set at *p* < 0.05. SDR: skull density ratio.

**Figure 6:**
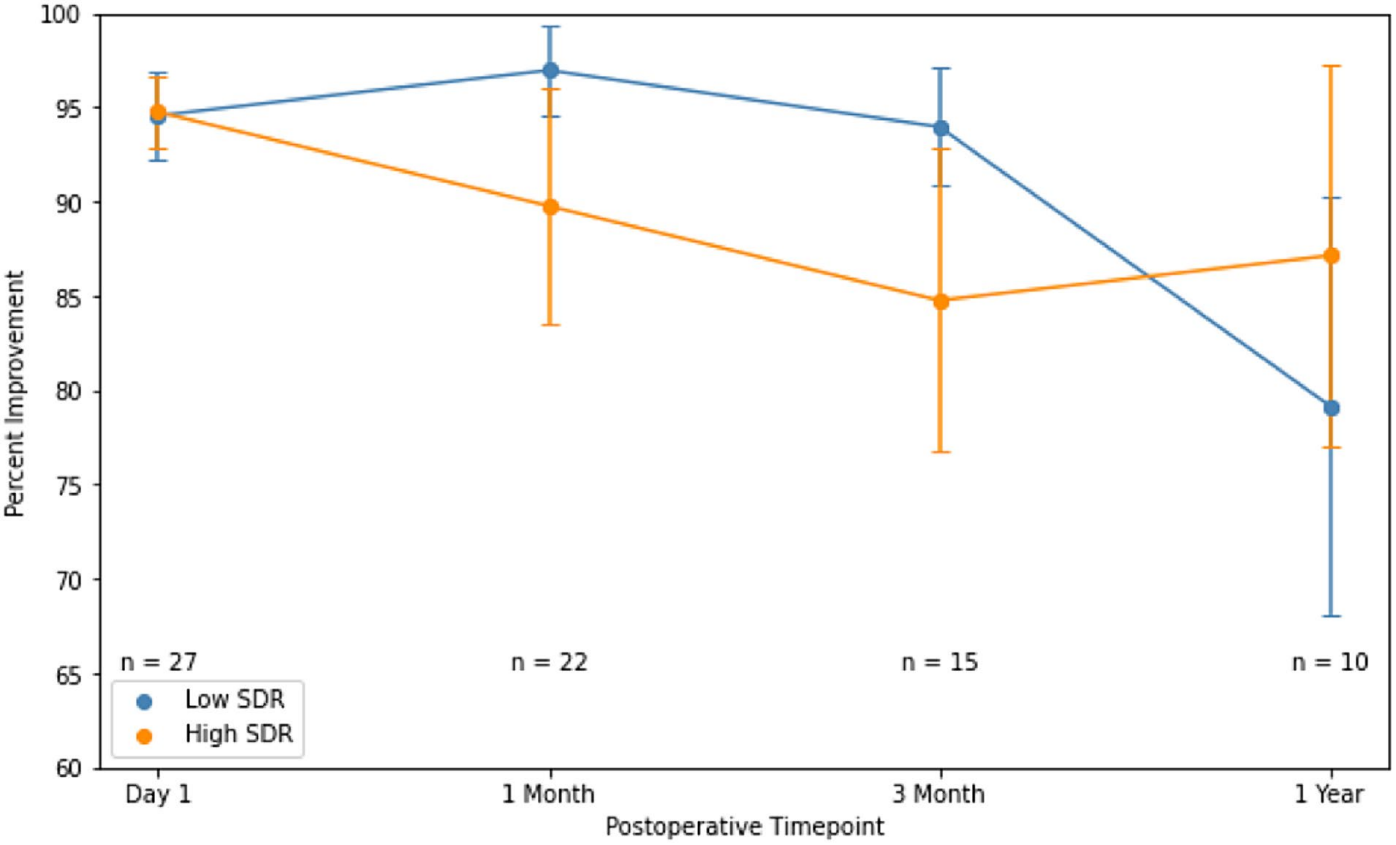
Percent improvement in essential tremor (intention + posture FTM scores) for patients at various timepoints after MRgFUS for essential tremor comparing case-matched cohorts with low SDR (*n* = 28) and high SDR (*n* = 28). Populations compared using independent *t*-tests. Significance set at *p* < 0.05. SDR: skull density ratio.

Absolute intention + posture FTM scores between low-SDR and high-SDR cohorts were not significantly different at every follow-up time point (Figure 5). Percent improvements in intention + posture FTM scores relative to baseline preoperative scores between low-SDR and high-SDR cohorts were also not significantly different at every follow-up time point (Figure 6). Regarding adverse events, there were significant differences between low-SDR and high-SDR cohorts in postoperative day 1 imbalance (low-SDR: 29.6%; high-SDR: 63.0%; *n* = 27; *p* = 0.03) and postoperative month 3 imbalance (low-SDR: 0%; high-SDR: 33.3%; *n* = 15; *p* = 0.05).

### 3.3 Illustrative case

A 74-year-old right-handed female presented with essential tremor, diagnosed 30 years prior. On presentation, she reported difficulty with buttons, make-up, writing, and inability to use a keyboard. At the time of presentation, she stated she no longer ate in public. She had tried a variety of medications but still had persistent tremor. She was on propranolol, which was initially very helpful when she started it 20 years prior but had lost most of its effect despite high dosing. On exam, she had full strength and normal gait with no evidence of bradykinesia or increased tone. She had mild head and vocal tremor with no rest tremor, as well as a moderate postural tremor (right greater than left) with an inability to draw a spiral or write legibly (rated head 1/4, vocal 1/4, postural 2/4, intentional 4/4). CT revealed an SDR of 0.33. After a discussion on the implications of her low SDR, she agreed to proceed with left-sided MRgFUS thalamotomy for treatment of right-sided tremor.

During the procedure, other than severe headache, no complications ensued. She had complete tremor abolition immediately after the procedure. At day 1, she had continued abolition of tremor in the right hand as well as improved vocal tremor (from 1/4 to 0/4) with no side effects. At 1 week, she maintained tremor response, but had a slightly unsteady gait and some fatigue. At 1 month, her fatigue resolved, and her gait was almost completely back to normal. At most recent follow-up of 1 year, she continued to demonstrate no tremor in the right hand with improvement in vocal tremor, and she felt her side effects had completely resolved.

## 4 Discussion

Overall, in this large single-center analysis, unilateral MRgFUS thalamotomy was feasible and effective in patients with SDR <0.40. There were some important differences to consider between patients with low and high SDR, including some significant differences in sonication parameters, lesion volumes, and side effects. While patients with low SDR had slightly lower tremor control, there was no significant difference at any timepoint. In the case-matched analysis, patients with high and low SDR showed similar tremor outcomes, although the patient populations were smaller.

The overall analysis showed that patients with low SDR required greater maximum power, energy, and duration and reached lower maximum temperature. This has been previously demonstrated, as patients with lower SDR have lower heating efficiency (8–11, 21, 23, 24). A potential cofounder is skull thickness, which has also been shown to impact ultrasound energy efficiency (25). Although increasing sonication power and duration for lower SDR patients has the potential to overheat the skin and skull (9), no significant adverse effects were seen with these parameter changes. Nevertheless, patients with low SDR can experience more side effects during treatment, such as severe headache or nausea and vomiting, which may prohibit successive sonications and prevent tremor from being completely abolished. Anecdotally, the surgeons here confirm that higher treatment parameters result in much higher discomfort during the procedure, although no formal analysis was performed.

Thalamotomy lesion volumes were significantly smaller in patients with lower SDR. This trend has been previously demonstrated by Vetkas et al. (19), although the difference was not significant in their population (150 ± 94 mm^3^ vs. 131 ± 98 mm^3^, *p* = 0.401). The lesions created in that study were much smaller than in our population, which may explain the difference. Reasonably sized lesions can be created in patients with low SDR, but this becomes more difficult as the lesion gets larger. We also found, as demonstrated in recent reports by D’Souza et al. (11) and Vetkas et al. (19), that low SDR patients had slightly lower side effect profiles. We also demonstrated no difference in long-term tremor control. The effect of SDR on tremor outcomes and side effects has been debated, but the significance here is likely a consequence of volume discrepancies (8, 11–13, 16, 26, 27). The case-matched analysis also supports that there are no major differences between the tremor outcomes in the two populations, although there were fewer patients in this analysis. Additionally, because population sizes diminished at later follow-up timepoints, tremor outcomes at long-term follow-up cannot be as confidently assessed.

The findings here may seem to suggest that contrary to current thinking, patients with lower SDR are better candidates given the insignificantly lower tremor control and lower side effect profile. However, it is important to consider that three patients with low SDR were excluded given the inability to create a satisfactory lesion. Patients with low SDR need to be counseled properly on the requirement for longer, potentially more uncomfortable sonications that still may result in treatment failure. If a lesion is able to be created, the outcomes will be more closely correlated with lesion size rather than SDR. Additionally, practitioners should be wary that it is easier to make a lesion that is too large in patients with higher SDR, potentially leading to a greater side effect profile.

The main limitation, as aforementioned, is the loss of patient follow-up at later timepoints. There was no clear explanation for the loss to follow up. Additionally, it is difficult to compare our population to other studies because MRgFUS technique, goal lesion size, and subjective follow-up measures can vary between institutions. Another limitation of our dataset is the omission of the FTM disability subscale, which measures the impact of tremor on activities of daily living and is therefore an informative marker of treatment success. Finally, although SDR is the main measure used to exclude patients from undergoing MRgFUS thalamotomy, the skull features that impact effective lesioning is likely more complex, including volume, shape, and presence of hyperostosis and skull thickness. Future studies should look into these characteristics to get a more comprehensive analysis of factors affecting lesioning.

## 5 Conclusion

In summary, ET patients with SDR <0.40 can be safely and effectively treated with MRgFUS. Sonication parameters need to be adjusted accordingly to create effective lesions, including higher energy, power, and duration. Maximum temperatures may be lower in patients with low SDR than in patients with high SDR resulting in smaller thalamotomy lesion volumes on postoperative MR imaging. The smaller lesion volume may explain lower adverse event profiles, but tremor control appears to be comparable. As MRgFUS technology expands to include additional patient populations and indications, patients with low SDR can be considered for treatment but should be advised on potential treatment discomfort and slight outcome differences.

## Data availability statement

The raw data supporting the conclusions of this article will be made available by the authors, without undue reservation.

## Ethics statement

The studies involving humans were approved by Brigham and Women’s Hospital Institutional Review Board. The studies were conducted in accordance with the local legislation and institutional requirements. Written informed consent for participation was not required from the participants or the participants' legal guardians/next of kin due to the retrospective nature of the study.

## Author contributions

PN: Conceptualization, Investigation, Writing – review & editing, Writing – original draft, Methodology, Data curation. SB: Writing – review & editing, Writing – original draft, Visualization, Software, Methodology, Investigation, Formal Analysis, Data curation. MC: Writing – review & editing, Methodology, Data curation, Conceptualization. GC: Writing – review & editing, Supervision, Resources, Project administration, Conceptualization.

## Footnotes

^1^ https://www.slicer.org

## Supplementary material

SUPPLEMENTARY FIGURE S1 Violin plots of sonication number, treatment sonications with energy > 5000 J, mean maximum power, mean maximum energy, mean maximum sonication duration, and mean maximum temperature between low-SDR (*n* = 28), medium-SDR (*n* = 202) high-SDR (*n* = 37) cohorts. Continuous variables were analyzed with independent *t*-tests. Significance set at *p* < 0.05. SDR: skull density ratio.

SUPPLEMENTAL FIGURE S2 Percent improvement in essential tremor (intention + posture FTM scores) for patients at various timepoints after MRgFUS for essential tremor comparing all patients with low SDR (*n* = 28), medium SDR (*n* = 202), and high SDR (*n* = 37). Populations were compared using independent ANOVAs. Significance set at *p* < 0.05. SDR: skull density ratio.
