# Supplementary figures and images for: Magnetic resonance-guided focused ultrasound thalamotomy for essential tremor patients with low skull density ratio: a case-matched analysis
Source: Front Neurol. 2024 Apr 22;15:1370574. doi: 10.3389/fneur.2024.1370574 (PMC11071343; doi:10.3389/fneur.2024.1370574)

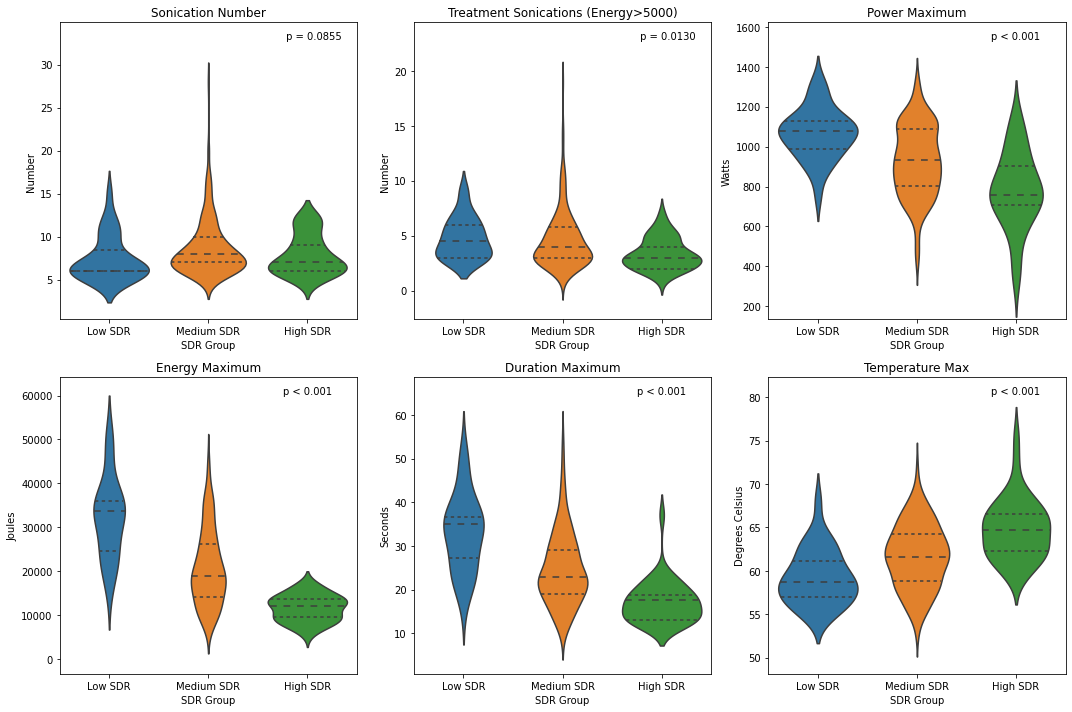

Supplement: SUPPLEMENTARY FIGURE S1 — Violin plots of sonication number, treatment sonications with energy > 5000 J, mean maximum power, mean maximum energy, mean maximum sonication duration, and mean maximum temperature between low-SDR (n = 28), medium-SDR (n = 202) high-SDR (n = 37) cohorts. Continuous variables were analyzed with independent t-tests. Significance set at p < 0.05. SDR: skull density ratio. [file Image_1.tiff]

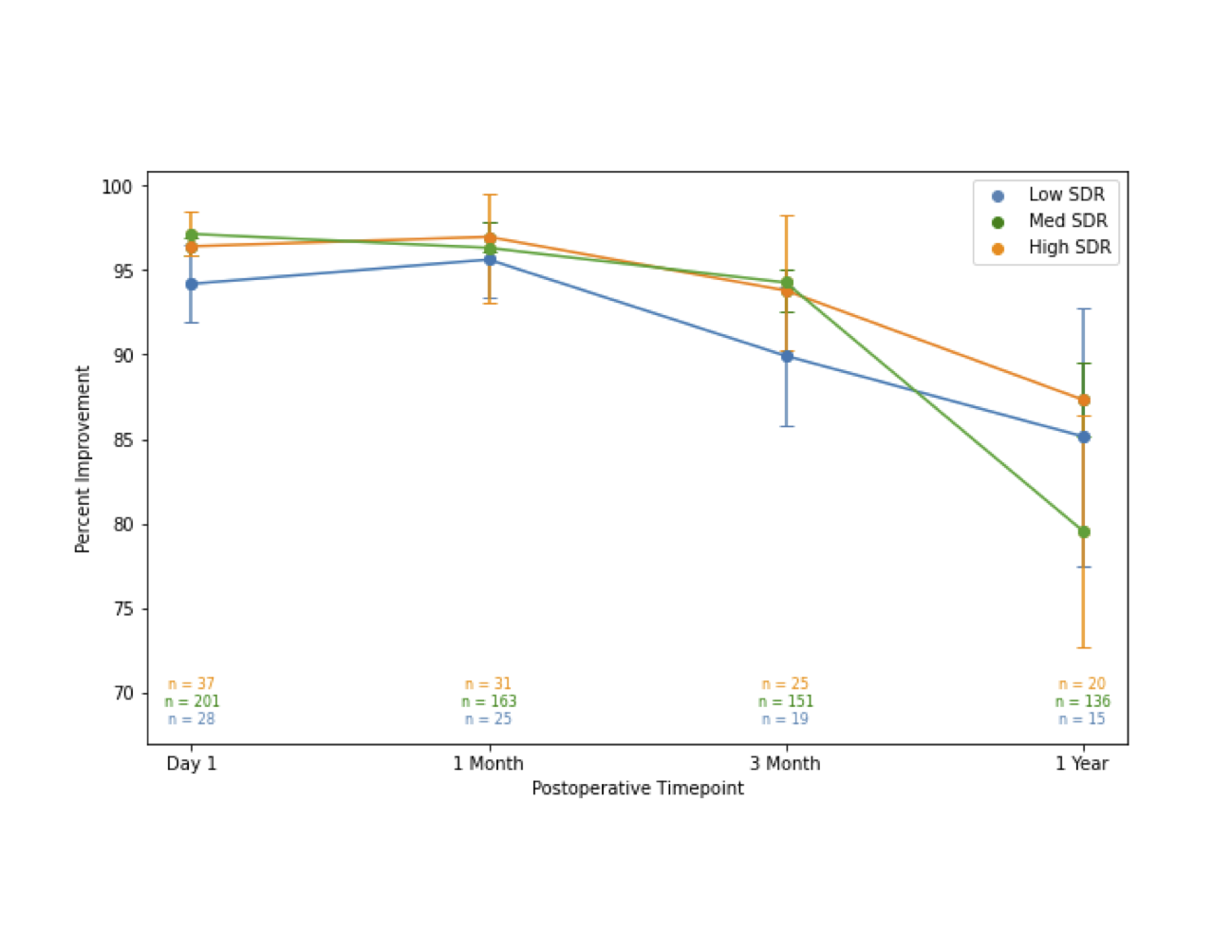

Supplement: SUPPLEMENTAL FIGURE S2 — Percent improvement in essential tremor (intention + posture FTM scores) for patients at various timepoints after MRgFUS for essential tremor comparing all patients with low SDR (n = 28), medium SDR (n = 202), and high SDR (n = 37). Populations were compared using independent ANOVAs. Significance set at p < 0.05. SDR: skull density ratio. [file Image_2.TIFF]
